# Artificial Intelligence Approaches for Geographic Atrophy Segmentation: A Systematic Review and Meta-Analysis

**DOI:** 10.3390/bioengineering12050475

**Published:** 2025-04-30

**Authors:** Aikaterini Chatzara, Eirini Maliagkani, Dimitra Mitsopoulou, Andreas Katsimpris, Ioannis D. Apostolopoulos, Elpiniki Papageorgiou, Ilias Georgalas

**Affiliations:** 1 1st Department of Ophthalmology, G. Gennimatas General Hospital, National and Kapodistrian University of Athens, 11527 Athens, Greece; katxat60@gmail.com (A.C.); eirini.maliagani@gmail.com (E.M.); igeorgalas@yahoo.com (I.G.); 2 Eye Unit, University Hospital Southampton, Southampton SO16 6HU, UK; dimits96@gmail.com; 3 Princess Alexandra Eye Pavilion, University of Edinburgh, Edinburgh EH3 9HA, UK; katsimprisandreas@hotmail.com; 4 ACTA Lab, Department of Energy Systems, University of Thessaly, Gaiopolis Campus, 41500 Larisa, Greece; ece7216@upnet.gr

**Keywords:** artificial intelligence, deep learning, convolutional neural networks, segmentation, geographic atrophy, age-related macular degeneration, retinal imaging, ophthalmology

## Abstract

Geographic atrophy (GA) is a progressive retinal disease associated with late-stage age-related macular degeneration (AMD), a significant cause of visual impairment in senior adults. GA lesion segmentation is important for disease monitoring in clinical trials and routine ophthalmic practice; however, its manual delineation is time-consuming, laborious, and subject to inter-grader variability. The use of artificial intelligence (AI) is rapidly expanding within the medical field and could potentially improve accuracy while reducing the workload by facilitating this task. This systematic review evaluates the performance of AI algorithms for GA segmentation and highlights their key limitations from the literature. Five databases and two registries were searched from inception until 23 March 2024, following the PRISMA methodology. Twenty-four studies met the prespecified eligibility criteria, and fifteen were included in this meta-analysis. The pooled Dice similarity coefficient (DSC) was 0.91 (95% CI 0.88–0.95), signifying a high agreement between the reference standards and model predictions. The risk of bias and reporting quality were assessed using QUADAS-2 and CLAIM tools. This review provides a comprehensive evaluation of AI applications for GA segmentation and identifies areas for improvement. The findings support the potential of AI to enhance clinical workflows and highlight pathways for improved future models that could bridge the gap between research settings and real-world clinical practice.

## 1. Introduction

### 1.1. Age-Related Macular Degeneration—Geographic Atrophy Definition

Age-related macular degeneration (AMD) is a chronic degenerative disease of the retina and a leading cause of blindness in older adults. The prevalence of AMD is expected to rise due to the rapidly aging global population, making it a significant public health issue in the coming decades [1]. AMD is classified into two main forms: dry (nonexudative) AMD and wet (exudative or neovascular) AMD. Geographic atrophy (GA) is an advanced form of dry AMD and is characterized by the gradual loss of the retinal pigment epithelium (RPE), photoreceptors, and underlying choriocapillaris in roughly circular areas in the posterior pole, leading to severe central vision loss when the fovea is involved [2,3].

The term “geographic atrophy” was first reported in association with “senile macular degeneration” by J. Donald and M. Gass in 1972 [4]. Various classification systems were developed in the following years that defined GA as well-circumscribed hypopigmented retinal lesions exposing choroidal vessels, with a minimum diameter of 175 μm on color fundus imaging [5]. However, as modern imaging techniques have emerged, a more appropriate nomenclature has been proposed in order to catch up with the improved visualization methods and provide standardized terminology. The Classification of Atrophy Meetings (CAM*) consensus introduced four new terms to further classify AMD-related atrophy based on the optical coherence tomography (OCT) findings. Complete RPE and outer retinal atrophy (cRORA) is defined as an area of hypertransmission and RPE attenuation of >250 μm in the greatest diameter with overlying degeneration of the photoreceptor layer and no signs of an RPE tear. Incomplete RPE and outer retinal atrophy (iRORA), complete outer retinal atrophy (cORA), and incomplete outer retinal atrophy (iORA) describe earlier stages of the atrophic process in AMD [6]. Although the term GA is considered a subset of cRORA, it is still widely used in routine clinical practice and in the literature and is strongly associated with end-stage AMD atrophy, so, for the purpose of this review, we employed both terms to access a more comprehensive range of the literature [6]. Although OCT has been proposed as the “gold standard” imaging modality for GA identification and staging, other visualization approaches, like fundus autofluorescence (FAF), near-infrared reflectance (NIR), fluorescein angiography (FA), and color fundus photography (CFP), still offer useful clinical information and are routinely encountered in many clinical settings [6].

The early detection of GA is vital because it allows for timely referral and intervention before extensive retinal damage occurs [7]. Additionally, GA lesions often expand slowly, with enlargement rates ranging from 0.53 to 2.79 mm^2^ per year in the literature, making disease progression monitoring challenging [8]. At the same time, several clinical trials chose anatomic endpoints, like the GA lesion growth rate, to assess the efficacy of emerging therapies [9]. In order to track those subtle atrophic changes, assist clinicians in timely GA detection, and quantify lesion shape and size, accurate and reliable segmentation techniques are needed.

### 1.2. Geographic Atrophy Segmentation

Image segmentation is a valuable component of medical image analysis and involves the process of separating an image into multiple components and isolating the regions of interest (ROIs) [10]. In the context of GA segmentation, it relies on lesion partitioning from the surrounding healthy retinal tissue, which is crucial for monitoring lesions across different visits, predicting disease progression, and optimizing insights from multimodal imaging sources.

Based on the segmentation approach employed, it can be classified as manual, semi-automatic, or fully automatic [11]. Manual segmentation involving a human grader has traditionally been the gold standard (or “ground truth”—GT) due to its wide clinical application and an expert’s ability to interpret the fine details; however, it requires significant time and effort and is subject to variability among specialists or imaging modalities [12,13]. Semi-automatic segmentation combines manual annotations with computational assistance, with varying degrees of user intervention [11]. Lastly, fully automatic image segmentation has long been in the research spotlight with the hope of providing accurate, fast, and consistent results with minimal-to-no human interaction. Region-based methods, deformable models, and other approaches have been used for this purpose [14], as well as artificial intelligence (AI) techniques, which have received considerable attention as promising automatic segmentation tools for achieving impressive speed, consistency, and accuracy [15].

### 1.3. Artificial Intelligence

AI is a broad field encompassing various techniques that enable computers to mimic human intelligence and perform advanced tasks, like learning, problem-solving, and decision-making. In medical imaging, AI plays a crucial role in automating tasks, improving efficiency and precision, and providing insights that may not be readily apparent to human users [16]. Deep learning (DL) is a subfield of AI that utilizes deep structures, such as artificial neural networks with multiple layers (adding “depth”), to automatically learn from complex data. This data-driven approach allows DL models to achieve exceptional performance on various image analysis tasks by directly extracting the features from the raw image data, outclassing predecessor AI subfields like machine learning (ML) [17].

It is no surprise that AI algorithms have seen extensive application in ophthalmology. In the context of AMD, numerous models have been developed to tackle various aspects of medical image analysis, including disease detection, classification, segmentation, treatment response, progression, and prediction [18]. As these AI techniques have evolved, their application has become increasingly focused on GA assessment, particularly the segmentation of the relevant lesions, enabling a more precise delineation of the atrophic boundaries [15]. Several clinical trials investigating complement inhibitors for GA treatment have used the lesion area growth as the primary endpoint, and AI models that can segment GA lesions have served as valuable tools for monitoring patients and tracking these endpoints efficiently, often with pixel-level accuracy [19]. Moreover, the accurate contouring of GA lesion boundaries via automatic approaches is crucial for computer-aided diagnosis (CAD) and enhanced image interpretation, reduced clinical workload, and supported decision-making [20]. In line with these advancements, the first AI-driven approach for GA analysis to be certified under the European Union Medical Device Regulation is the RetInSight GA Monitor, a clinical decision support system (DSS) developed for integration into routine practice and patient monitoring [21].

The development of AI models for such tasks is a stepwise process that generally follows an established pipeline. A dataset with appropriate images is gathered and is later split into training, validation, and internal test sets. The first set is used to build the model with the optimal parameters that balance performance with overfitting, as determined by multiple iterations of the validation set. Then, an unseen test set is used for the evaluation of the final model’s performance and an assessment of generalizability. Very often, an independent, external test set is employed to assess its performance across different populations, robustness, and real-world applicability [22]. However, we should note that this process is a general outline of model development that may not apply to all AI algorithms (i.e., unsupervised models).

An integral part of AI algorithm development is the evaluation of the model outputs. Apart from a visual evaluation of the segmentation results, which offers a qualitative reassurance of a model’s performance, measurable indices must be employed in order to quantify and scale the performance of AI models. The most common metric for the evaluation of image segmentation outputs is the Dice similarity coefficient (DSC), which measures the correspondence between model outputs and expert annotations [15]. Other important metrics include the F1 score, accuracy, precision, positive predictive value (PPV), sensitivity (recall), specificity, absolute area difference (AAD), area under the curve (AUC), intersection over union (IoU), and overlap ratio (OR) [23,24,25]. These metrics can not only objectively assess the segmentation results and aid in comparisons of models, but also assist in parameter fine-tuning, appropriate algorithm selection, and qualitative dataset analysis [23].

It is also worth mentioning that the rapid development of AI-driven medical image analysis has quickly led to its expansion into decision-making processes, like diagnosis and treatment planning. However, even the most highly accurate and powerful algorithms cannot be integrated into clinical routines unless they provide a justification of their results, making them understandable by human users. Transparency and interpretability are essential before trusting a model with critical decision-making tasks, which is why the concept of explainable AΙ (XAI) has emerged to describe all the efforts and methodologies aimed at developing comprehensible and reliable AI systems [26]. To further address this issue, the European Union established the AI Act (AIA) in 2024, requiring healthcare AI DSSs to be explainable, either intrinsically or extrinsically, providing insights into the causal relationships between the inputs and outputs to enhance trustworthiness [27].

Despite the challenges arising along the way, AI continues to evolve at an unprecedented pace, reshaping ophthalmology, a specialty that is inherently dependent on medical imaging technologies. Within the vast field of AMD, GA segmentation holds great potential for AI applications, but this topic has yet to be addressed in a focused, comprehensive manner. In this work, we present a systematic review of the current literature on AI applications for GA lesion segmentation, charting the methodologies, datasets, performance metrics, and limitations of the AI algorithms used in this field. We critically analyze the various approaches with the aim of providing a clear picture of the state-of-the-art AI-driven segmentation methods for GA and identifying the gaps in the literature where further research is needed. This work serves as a foundation for advancing the application of AI technologies in ophthalmology and contributes to the growing body of knowledge about medical image analysis. To the best of our knowledge, no other systematic review has yet to address the concept of GA segmentation via AI methods, and our ambition is to provide confident insights into this rapidly evolving field while cautiously examining its potential for further development and clinical applicability.

## 2. Materials and Methods

### 2.1. Research Question

What are the accuracy and efficacy of AI tools for the segmentation of geographic atrophy lesions among ophthalmic imaging modalities?

### 2.2. Eligibility Criteria

This systematic review and meta-analysis was designed and conducted in accordance with the PRISMA guidelines (Preferred Reporting Items for Systematic Reviews and Meta-Analyses) [28], with the PRISMA checklist provided in Supplementary Table S1. A protocol was developed, but not registered, using the PICOS (Population, Intervention, Comparison, Outcomes, and Study Design) framework (Table 1) to define eligibility and address the research question [29]. To ensure a rigorous selection process, we established detailed inclusion and exclusion criteria, presented in Table 2.

### 2.3. Search Strategy and Study Selection

A systematic search was conducted of MEDLINE (via PubMed), Scopus, Google Scholar, Web of Science, Cochrane Library, ClinicalTrials.gov, and World Health Organization’s (WHO’s) International Clinical Trials Registry Platform (ICTRP) by two independent reviewers (A.C. and D.M.). Searches covered all available records until 23 March 2024. Since GA is often considered as late-stage AMD, we opted for broad, inclusive search algorithms that encompassed AI applications to AMD analysis in general, with the purpose of maximizing the eligible study results. The following key terms were used in various combinations: “deep learning”, “machine learning”, “artificial intelligence”, “AI”, “algorithm”, “late age-related macular degeneration”, “cRORA”, “geographic atrophy”, “GA”, and “automated segmentation”. Each reviewer (A.C. and D.M.) reassessed the suitability of the search strategy via multiple iterations until reaching a final consensus aligned with the research objective. The finalized search algorithms were then used for manual searches of each nominated database or registry. Detailed search algorithms and strategy are provided in Supplementary Table S2.

The retrieved literature was uploaded to EndNote (version x21.2) for efficient reference management. After automatic deduplication [30], the remaining papers were uploaded to the online reviewing platform “Rayyan” for accurate title and abstract screening [31]. The two reviewers (A.C. and D.M.) performed title and abstract screening blindly and independently, evaluating available literature against predefined inclusion and exclusion criteria (Table 2). Studies unrelated to AI applications for AMD/GA analysis were marked as “excluded”, while those with low/medium or high relevance were marked as “maybe” or “included”, respectively. Reviewer discrepancies were resolved through discussion, and the remaining records were re-exported to EndNote for full-text retrieval. A double, independent, comprehensive full-text screening was then performed, with documentation of the main exclusion reasons. Any disagreements were resolved by consensus, and the final group of included studies proceeded to the data extraction and risk-of-bias assessment phases.

### 2.4. Quality Assessment

To assess the quality of the included studies, two reviewers (A.C. and D.M.) independently applied the Quality Assessment of the Diagnostic Accuracy Studies-2 (QUADAS-2) [32] and the Checklist for Artificial Intelligence in Medical Imaging (CLAIM) [33] tools. Discrepancies were resolved through consensus with a senior researcher (E.M.). The combination of QUADAS-2 for diagnostic quality and CLAIM for reporting quality ensured that both the reliability of the results and the transparency of AI-specific methodologies were thoroughly assessed in a standardized and targeted manner.

The QUADAS-2 scale assesses risk of bias across four domains (patient selection, index test, reference standard, and flow and timing) and evaluates applicability concerns for three domains (patient selection, index test, and reference standard). The responses for each item are classified into five categories (i.e., yes, no, low, high, or unclear risk) according to specific questions within each component. The scale was used unmodified and regarding *patient selection*, emphasis was placed on ensuring a well-defined population of GA eyes without inappropriate exclusion of ambiguous or difficult-to-diagnose cases. The *index test* domain focused on the detail of description of the AI algorithm used for segmenting GA lesions with different ophthalmic imaging modalities. The *reference standard* domain assessed the reliability of the reference standard via manual delineation or other validated methods. Finally, the *flow and timing* domain evaluated the transparency, coherence, and reproducibility of the research process and patient management. Applicability concerns for each of the first 3 domains were rated for their relevance and agreement with the research question.

CLAIM offers a best practice checklist to promote transparency and reproducibility of medical imaging AI research. It consists of 44 items across key domains, including study design, dataset characteristics, reference standard, AI methodology, evaluation, and model performance. Each CLAIM item has three options: yes, no, and not applicable (N/A). “Yes” is assigned when all or most checklist requirements are met, allowing for minor omissions that do not affect reliability; “No” is assigned when key elements are missing, impacting quality, clarity, or replicability; and “Not Applicable” is assigned when an item is irrelevant to a study’s characteristics. We strictly adhered to CLAIM item directions, assessing each paper based on the specific structure and sectioning mandated by the checklist.

### 2.5. Data Extraction

Double independent extraction with mutual verification was performed by two reviewers (A.C and D.M.), with recorded information entered into a predefined Excel spreadsheet for later tabulation. Any discrepancies were resolved by a senior investigator (E.M.). A brief description of the categories of extracted data is presented in Table 3.

### 2.6. Statistical Analysis

We evaluated the performance of AI algorithms for the analysis of geographic atrophy using the DSC, a commonly used metric for assessing the overlap between predicted and reference segmentations. For the meta-analysis, we required the mean DSC values with their corresponding standard deviations (SDs) or 95% confidence intervals (CIs).

A random-effects model was employed to estimate the pooled DSC values, accounting for variability across the included studies. Forest plots were generated to visualize the individual studies’ effect sizes and their overall pooled performance. Heterogeneity among studies was assessed using the Cochrane Q test and the Higgins I^2^ statistic. An I^2^ value of 25–50% was considered low-to-moderate heterogeneity, while values exceeding 75% indicated substantial heterogeneity. A *p*-value of <0.05 on the Cochrane Q test was considered indicative of statistically significant heterogeneity.

All statistical analyses were conducted using R Statistical Software (v4.4.0; R Core Team 2024), and a two-sided *p*-value of <0.05 was deemed statistically significant.

## 3. Results

### 3.1. Study Selection

**Figure 1:**
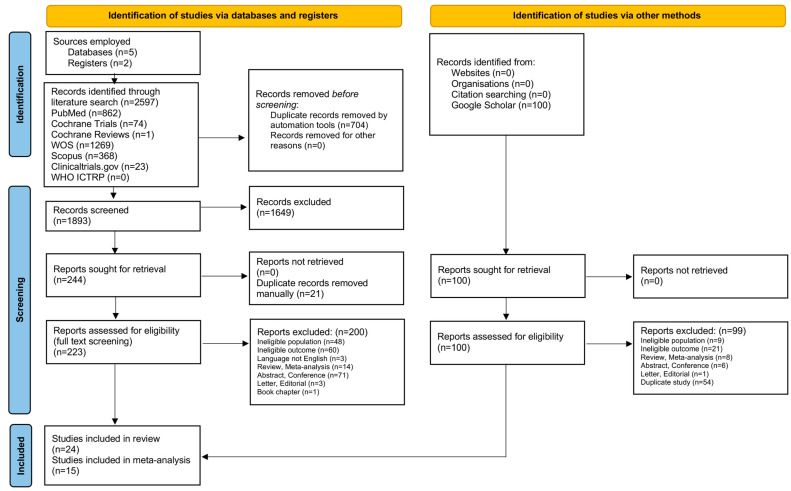
PRISMA flowchart of study selection process.

Our initial literature search yielded 2597 studies in total. After duplicate removal in EndNote, 1893 studies remained for title and abstract screening, leading to the exclusion of 1649 records, mainly due to an ineligible population (e.g., studies conducted in people without AMD) or ineligible intervention (e.g., AMD analysis without the use of AI tools). Among the 244 remaining records, we identified and manually removed 21 duplicates, ensuring consistency across the title, year, author, abstract, journal, and DOI. At this stage, we also performed a gray literature search of Google Scholar, adding the first 100 results to our final cohort. After a double, independent, full-text screening, 24 studies met all the eligibility criteria for the systematic review, and 15 were included in the meta-analysis. Forward and backward citation chasing (snowballing) did not yield additional studies. A detailed visual representation of the literature collection and screening process is provided in the PRISMA flowchart (Figure 1).

### 3.2. Risk of Bias, Applicability Concerns, and Reporting Quality

**Figure 2:**
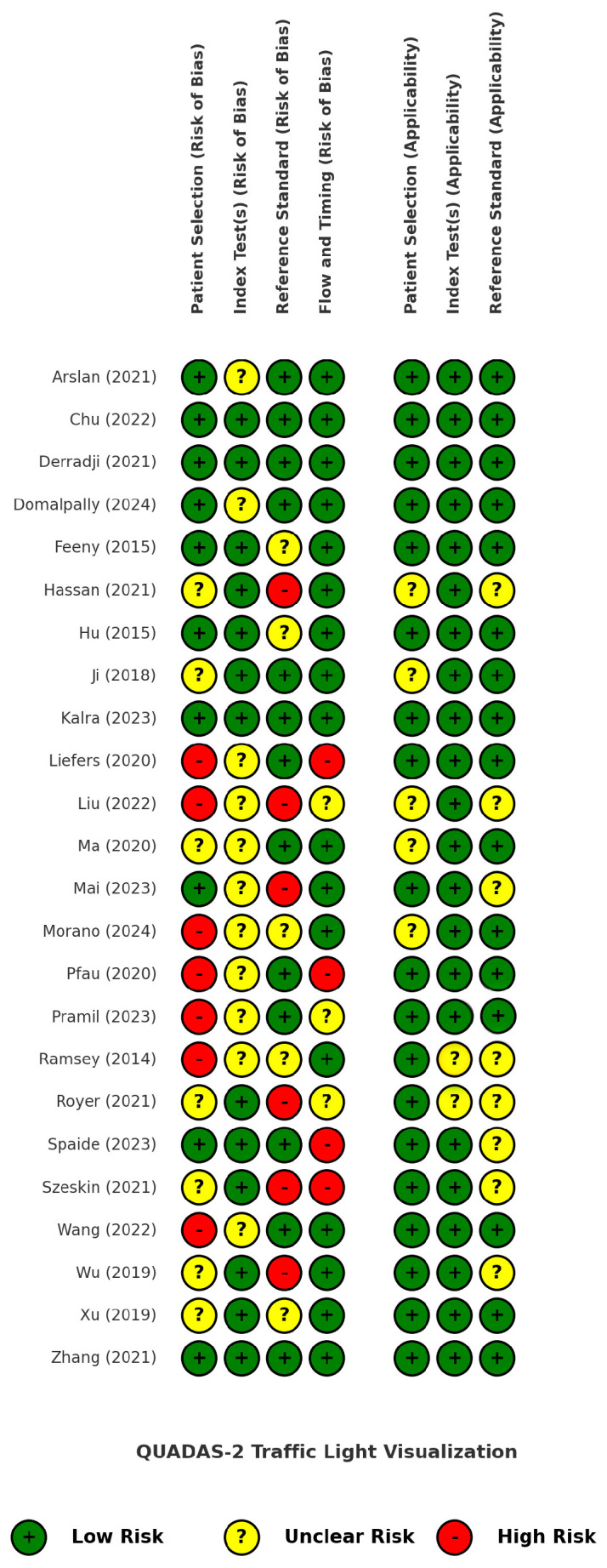
QUADAS-2 traffic light plot for risk of bias and applicability assessment of the included studies [34,35,36,37,38,39,40,41,42,43,44,45,46,47,48,49,50,51,52,53,54,55,56,57].

Figure 2 presents the findings of the quality assessment of the included studies using the QUADAS-2 tool, displayed as a traffic light plot for visual clarity. A more detailed analysis of the risk of bias and applicability concerns is provided in Supplementary Table S3.

Seven studies received a low score for the patient selection domain due to inappropriate exclusions, particularly related to poor image quality, unclear lesion borders, predefined lesion size thresholds, best-corrected visual acuity (BCVA) criteria, or non-random patient selection. These exclusions rejected a significant proportion of the patients (and their images) that are often encountered in everyday clinical practice, ultimately introducing bias and limiting real-world generalizability. For the index test domain, it was unclear whether a predefined threshold was used in 11 studies or if it was adjusted through trial-and-error or other non-standardized methods. Regarding the reference standard, potential sources of bias were identified in six studies, including the use of single-grader annotations, low or unreported inter-grader agreement, and a reliance on software or different imaging modalities as annotation proxies. The flow and timing domain showed a low risk of bias in 71% of the studies, but four studies had a high risk due to dissimilar reference standards among the patients (which can be partially attributed to the labor-intensive process of manual annotations) and the exclusion of patients along the study pipeline.

The applicability concerns were generally low, as the included studies highly matched our predefined GA population and AI intervention criteria and were meticulously selected from among a large pool of other automatic or semi-automatic GA segmentation studies, as dictated by our PICOS framework. However, the reference standard domain received an overall lower applicability score because it was often unclear whether the annotation process concluded in a GA ground truth that was reliable and consistent with our inclusion criteria.

**Figure 3:**
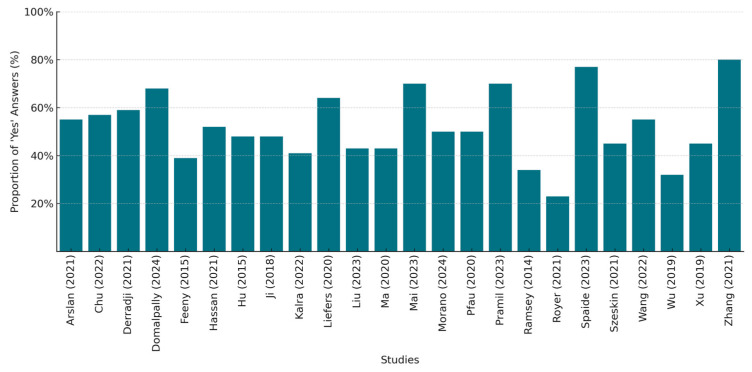
CLAIM checklist compliance per included study (%) [34,35,36,37,38,39,40,41,42,43,44,45,46,47,48,49,50,51,52,53,54,55,56,57].

Figure 3 presents a bar chart depicting the percentage compliance with the CLAIM checklist per study, by quantifying the number of positive answers for each of the 44 checklist items, while Supplementary Table S4 provides a detailed breakdown of the assessment results.

We strictly evaluated each study for its adherence to reporting guidelines and including the necessary information in the appropriate sections. There was a significant variation in their compliance, with the percentages ranging from approximately 23% to 80%. Only six studies achieved a compliance rate above 60%, with two of them exceeding 75%, indicating a better adherence to the reporting standards. In contrast, four studies had low compliance scores below 40%, suggesting possible gaps in their reporting quality. The common areas of poor performance included the justification of the testing sample size or reference standard selection, the inclusion of patient demographics and patient flow analysis, the description of the data anonymization techniques, the use of external testing, and the inclusion of the full study’s protocol links. Our overall evaluation aligns with the findings of other researchers, indicating that only a small percentage of the published literature on AI applications in medical imaging has adhered to the CLAIM checklist, providing documented evidence of self-reporting in accordance with the established standards, even among the studies that were published after the tool’s release [58].

### 3.3. Characteristics of Included Studies

**Figure 4:**
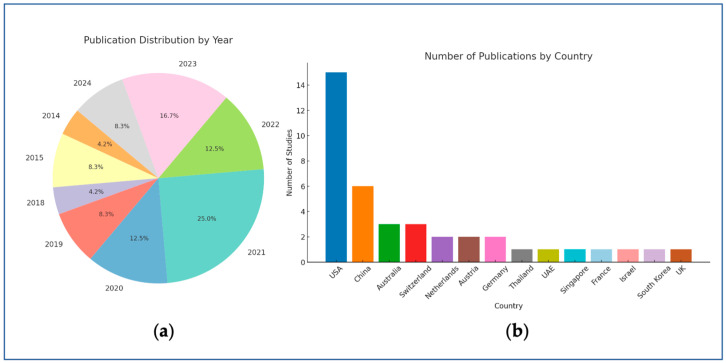
Publication trends by year (**a**) and country (**b**).

The 24 included studies, published between 2014 and 2024, show an overall increasing trend throughout the years (Figure 4a) and the vast geographical spread of AI research (Figure 4b). The studies’ authors are from 14 countries, with the largest contribution coming from the USA, followed by China.

Institutional or in-house datasets were used in 13 studies [34,35,36,37,38,39,40,41,42,43,44,45,46], while 3 other studies [47,48,49] relied on the frequently used datasets of Chen et al. [59]. The remaining studies [50,51,52,53,54,55,56] utilized subsets from different studies, randomized controlled trials (RCTs), or publicly available datasets and databases. One study [57] used both in-house patient data and a trial subcohort. Among the 24 studies analyzed, 19 [34,35,36,38,41,42,43,44,46,47,48,49,50,51,52,53,54,55,56] used datasets consisting of only GA images (with or without healthy controls), while the remaining studies [37,39,40,45,57] included datasets with a mix of GA cases and either earlier stages of AMD or other retinal diseases. Only five studies [35,36,39,44,56] adhered to the CAM* definitions of GA, incorporating the relevant prespecified imaging criteria into the model development process or distinguishing between the distinct atrophic stages.

We also observed that OCT and FAF were the two primary imaging modalities used in 15 [35,36,37,39,40,41,42,44,46,47,48,49,53,56,57] and 9 [34,38,42,45,46,50,53,54,55] of the studies, respectively, while CFPs were used in just 3 studies [51,52,54]. Only two studies [35,57] specified the use of swept-source optical coherence tomography (SS-OCT), while the rest utilized either spectral-domain optical coherence tomography (SD-OCT) [36,39,41,42,44,46,47,48,49,53,56] or did not clarify which type of OCT technology was employed [37,40]. A combination of modalities was identified in nine studies [41,42,44,46,47,48,53,54,55], three of which [41,47,48] used SD-OCT as the main imaging system and the corresponding FAF images as the GT annotation source, incorporating some degree of multimodality. Interestingly, three studies [37,39,52] used data from multiple manufacturers of the same imaging device type (various OCT or CFP models), providing valuable information on the generalizability across different technologies using the same modality, thus supporting broader applicability to a larger number of clinical settings.

The number of patients varied significantly, with one study recruiting only 10 patients [54], while another included 6953 patients with various retinal pathologies or healthy eyes, collected from an assemblage of different datasets [37]. Similarly, the dataset sizes fluctuated among the studies, ranging from 16 FAF images [38] to 100,266 OCT B-scans and 900 en face OCT images [39]. However, the datasets were not homogenous in type and contained different data formats (B-scan slices, images, OCT volumes, and cube scans), making absolute numerical comparisons futile and allowing for only broad stratification by size. The data partitioning in the training/validation/internal testing sets was not uniformly reported, with most of the included studies splitting the dataset either at the eye level, patient level, or image level, while four studies [43,46,48,54] provided no information on the division of the development data.

The ground-truth (reference standard) annotations were predominantly established manually by expert graders or ophthalmologists of varying expertise levels. We also documented several strategies to mitigate inter-grader variability and reduce the burden of manual high-volume annotation in a time- and cost-effective way. Those strategies include consensus-based grading, quantifying disagreement via several metrics (i.e., Cohen’s k, Intraclass Correlation Coefficient, Inter-grader DSC, etc.), delineating only selected B-scan slices from the OCT scan volumes, labeling the same images during two different sessions, and employing senior retina specialists to resolve discrepancies or correct the segmentation outcomes. One study used a previously validated ML model for retinal layer segmentation [39], while seven others [34,41,44,45,52,55,57] were assisted using segmentation software for distinct parts of the annotation process (RegionFinder, OCTAVO, Plex Elite Review, or an other non-commercial in-house software), often with a final reviewing and correction of the results by experts. The rest of the models relied solely on human-derived reference standards, with five studies [37,38,42,46,54] depending on single-grader annotations.

DL algorithms were the predominant approach in 87.5% of the studies, with convolutional neural networks (CNNs) employed in 19 studies [34,35,36,37,39,40,41,42,43,44,45,46,48,50,52,53,55,56,57]. U-Net and U-Net-like architectures were the most commonly adopted CNN variants, deployed with several modifications or adaptations in 12 studies [34,35,36,39,41,43,45,46,50,55,56,57]. Five studies did not rely on CNNs and utilized other custom ML/DL algorithms, like random forest classifiers [51], K-nearest neighbors (k-NN) [38], sparse autoencoders [47,49], or fuzzy c-means clustering [54].

While several studies included partially explainable features, like prediction maps or a feature importance analysis, comprehensive XAI techniques were reported in only five studies. These studies utilized class activation mapping (CAM)-based methods [40,48], saliency maps [48,50], probability maps [46], knowledge distillation-based anomaly localization heatmaps [40], and attention modules [45] to enhance the model’s post hoc interpretability.

Regarding the internal validation methods, k-fold cross-validation (k = 4, 5, or 8) was the most popular, encountered in 10 studies [34,36,38,41,44,45,48,50,52,57], followed by hold-out validation, which was applied in 8 studies [37,39,40,42,47,53,55,56]. At the same time, an external validation was conducted in 4 out of the 24 studies [41,50,52,56], with clinical study datasets being the most frequently employed. No real-world testing was performed in any of the included models.

The performance metrics varied across the studies, with the DSC and sensitivity (recall) being the most commonly reported, used in 17 [34,35,36,37,40,41,42,45,46,48,50,51,52,53,55,56,57] and 14 [34,35,36,37,38,39,41,43,44,45,51,54,56,57] studies, respectively, while the area under the precision-recall curve (AUPR), area under the receiver operating characteristic curve (AUROC), AUC, AAD, IoU, and F1 score were the least reported metrics.

The DSC values ranged from 0.680 to 0.978 for the internal testing and from 0.66 to 0.96 for the external testing. Among the four studies using external validation datasets, the performance tended to be higher compared to the internal validation results in three of the studies [41,50,56], suggesting that these models may have generalized well to the unseen data, or that the external datasets were more similar to the training data than expected. In contrast, the DSC in one study [52] remained almost unchanged between the internal and external testing datasets, but dropped significantly when non-GA cases were included in the unseen data cohort. The IoU and OR are two mathematically equivalent metrics for spatial overlap evaluation, offering a stricter assessment of over- or under-segmentation than the DSC [60]. These metrics were used in a total of seven studies [37,38,45,46,47,48,49], achieving a joint range of 0.606 to 0.9985.

**Figure 5:**
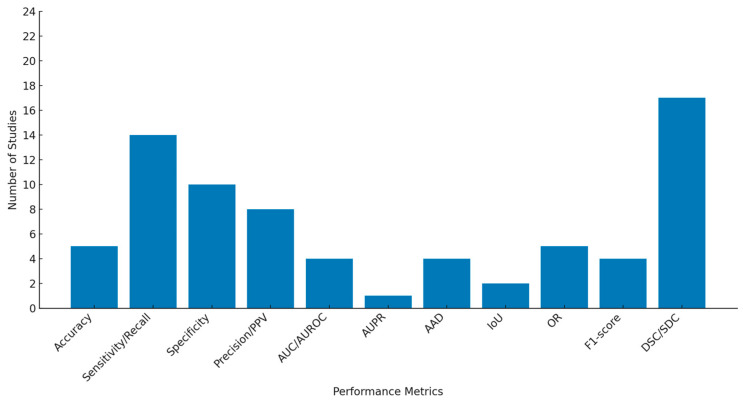
Distribution of performance metrics across included studies.

The sensitivity values ranged from 47% to 100%; however, this metric may refer to either the GA segmentation, GA detection, or binary pixel classification. Most of the studies incorporated classification-related metrics (apart from strict segmentation evaluations), providing valuable additional information on model performance. Similarly, the overall specificity varied between 42% and 100%, and the accuracy ranged from 82% to 98%. The distribution frequencies of all the pre-selected evaluation metrics are depicted in Figure 5.

Table 4 presents the basic characteristics of each study regarding the datasets, imaging modalities, and annotation methods. Table 5 focuses on the segmentation methods, validation approaches, and data partitioning, and Table 6 displays the algorithm architecture, explainability, and performance evaluation of each model.

### 3.4. Meta-Analysis Results

**Figure 6:**
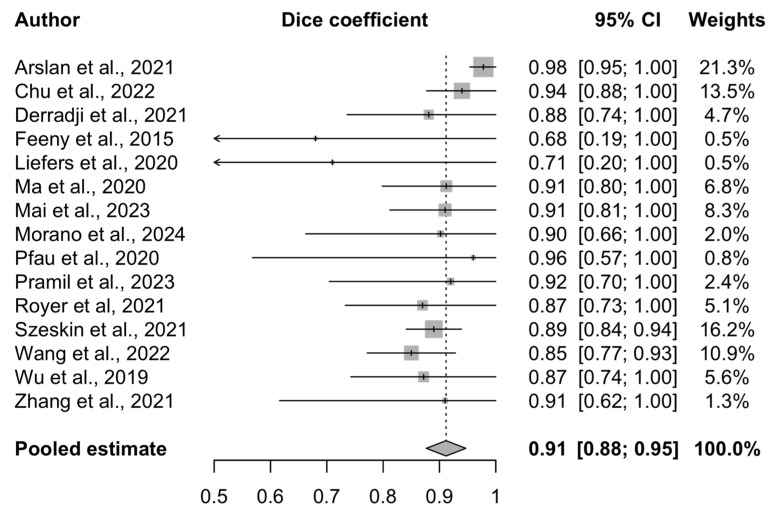
Forest plot of included studies [34,35,36,41,42,43,44,45,46,48,51,52,53,56,57] assessing AI model performance for geographic atrophy segmentation.

The performance of the 15 AI models included in this analysis is presented in the forest plot (Figure 6). The pooled DSC is 0.91 (95% CI 0.88–0.95), indicating a high agreement between the predicted and reference segmentations. This high degree of overlap suggests that the AI algorithms perform at a clinically meaningful level and may support patient monitoring or assist in decision-making, particularly in high-volume clinical environments.

A random-effects model was used, and study weights were calculated using the inverse-variance method. This approach incorporates both the within-study variance (reflecting the sample size and standard error) and between-study heterogeneity (tau^2^), ensuring that the more precise studies exert a proportionally greater influence on the pooled estimate.

The Cochrane Q test yielded a value of 23.66 (*p* = 0.05), and the Higgins I^2^ statistic was 40.8% (95% CI 0–67.9%), suggesting moderate heterogeneity across the included studies. This likely reflects the differences in the AI architectures, imaging modalities, dataset sizes, and annotation practices. The study quality was not used as a weighting factor in the meta-analysis; however, the risk of bias was assessed separately and is presented in the corresponding section.

## 4. Discussion

GA segmentation is a valuable AI-driven innovation in both retinal research and clinical ophthalmic practice. The present systematic review summarizes the current literature and provides meaningful conclusions that will aid in advancing this evolving field lying at the intersection of medicine and technology.

A previous review by other researchers of AI-based approaches to GA evaluation identified 18 studies addressing GA segmentation [15]. The present systematic review excluded traditional image processing techniques and collected 24 pure AI models for further analysis. To the best of our knowledge, this is the first systematic review and meta-analysis focused on AI algorithms for GA segmentation. This comprehensive synthesis of performance metrics and model development strategies captures the current state-of-the-art, examines key challenges and limitations in the field, and proposes future directions for developing robust and clinically applicable models. 

Although the earliest definitions of GA were based on CFP [5], only three studies [51,52,54] in our cohort presented AI models using this imaging modality, indicating that AI research is moving in parallel with newer imaging technologies that are better suited for GA assessment. The segmentation of atrophic lesions is suboptimal in CFP due to the low contrast or phenotypic variability [51], which allowed FAF to gain traction due to its inherent technological advantages, and ultimately become the preferred imaging modality in GA clinical trials [21]. Several of the studies included in our review highlight the notable differences in segmentation performance between CFP and FAF images. The literature generally indicates that CFP-based algorithms consistently show a lower performance due to media opacities, poor contrast, and variability in choroidal vessel presentation, which can hinder the clear delineation of atrophic lesions—even for expert graders [34,51,54]. In contrast, FAF provides a higher contrast between GA and the surrounding retina, enabling more accurate intensity-based segmentation [51]. However, FAF is not without limitations. Difficulties in assessing foveal involvement—due to natural autofluorescence suppression in the macula—and image artefacts, such as blurriness and shadowing, can impact segmentation accuracy [34,50,51]. Among the included studies, only one [54] directly compared segmentation outcomes using the same model applied to both CFP and FAF images, concluding that while FAF allowed for a more sensitive detection of GA, it introduced specific segmentation errors—particularly near the macular region—due to signal ambiguity. Future strategies combining CFP and FAF may enhance the delineation accuracy, particularly in parafoveal GA regions, by leveraging the complementary strengths of both modalities [51]. While most of the selected studies did not explicitly address the effect of the FAF wavelength on the segmentation performance, some reported reduced accuracy in the foveal regions, which may be partly attributed to macular pigment absorption—particularly relevant in short-wavelength (blue) FAF imaging acquired with confocal scanning laser ophthalmoscopy (cSLO) systems [34,54].

At the same time, the establishment of the CAM* criteria and the suggestion of OCT as the gold-standard modality for GA diagnosis and staging, along with its widespread clinical use, layer-by-layer analysis, and high sensitivity in the early atrophic stages [6], justify its dominance in our literature collection. As expected, all the included studies that adhered to the CAM* definitions and used OCT as the modality of choice were published after 2021, three years following the introduction of the consensus definitions in 2018 [6]. However, only five studies [35,36,39,44,56] followed the CAM* criteria, which may be attributed to the increased complexity of GA’s definition [4], along with the fact that the CAM* classification of GA subtypes relies exclusively on OCT B-scans, rendering it incompatible with models using FAF, CFP, or cSLO. Furthermore, studies have highlighted substantial inter-grader variability when the CAM* definitions are applied in real clinical settings, even among retina specialists, emphasizing the need for additional training before widespread implementation of the related terminology [61].

The majority of the included models were trained and evaluated on GA-exclusive datasets, likely to ensure precise segmentation performance without interference from other retinal pathologies. However, studies incorporating a broader range of conditions may provide insights into the models’ abilities to differentiate GA from other retinal abnormalities, which is crucial for real-world clinical applications. This highlights the trade-off between optimized segmentation performance in GA-only datasets and model generalizability in versatile datasets.

CNNs were the prevalent type of neural network architecture in 79.1% of the studies. They are widely used in many medical specialties for image localization tasks due to their efficient feature learning and extracting capabilities, which can often reach human-level accuracy [24]. U-Net, a well-established architecture for medical image analysis, was widely adopted in our literature cohort, either in its original, “vanilla” form [62], or one of its variants. Its simple structure, strong performance on small datasets, and ability to retain spatial details make it a popular choice for medical segmentation models [63].

The most frequently reported metric, the DSC, is considered the most suitable for evaluating the segmentation performance, as it quantifies the pixel-level degree of overlap between the predicted and ground-truth segmentation outputs [34]. Notably, when the DSC and F1 score—two numerically equivalent metrics that evaluate the spatial agreement between the predictions and reference standard [64]—are considered together, a combined total of 20 out of the 24 studies reported them, making overlap-based evaluation the predominant approach in assessing AI models for GA segmentation. The pooled DSC was 0.91 (0.88–0.95) among the studies included in this meta-analysis, indicating excellent AI model performance on GA segmentation, and suggesting its significant clinical and research promise. In everyday practice, however, there is significant variability in imaging results, lesion phenotypes, and patient characteristics. Therefore, even the strongest of models require rigorous validation before being widely adopted in healthcare. Identifying the weaknesses in current AI research on GA segmentation may facilitate the development of improved algorithms.

A commonly detected limitation was the relatively small dataset sizes, in terms of the number of images, patients, or eyes. Three studies [38,43,54] recruited 16 patients or fewer, and the smallest dataset sizes for each commonly used modality were noted in the three studies: one with 56 OCT volumes [46], one with 16 FAF images [38], and one with 26 FAF-CFP image pairs [54]. In general, the sample sizes have shown an increasing trend throughout the years, in line with the progress of AI technology and the need for scalable datasets for optimal performance; however, access to larger datasets is still limited. The scarcity of labeled GA imaging data could be addressed through several strategies, such as transfer learning, data augmentation, and synthetic data generation [65]. Additionally, collaboration between institutions using secure and nontraceable data-sharing frameworks could facilitate the collection of large, diverse datasets for robust AI model development [66].

In addition to dataset size, limitations related to GT quality and model validation practices were also observed. There was occasional reliance on single-grader GT establishment and a lack of inter-rater variability assessment, as five models [37,38,42,46,54] used annotations derived from one grader per image and three more studies [40,43,50] shared no information on the number of human graders involved. Six additional studies [35,39,45,49,51,53] utilized at least two graders/reviewers but did not calculate the inter- or intra-observer variability to assess the reliability of the GT. Manual delineations, across the medical imaging field, are known to be laborious and error-prone due to inter-rater disagreements, stressing the need for the measurable evaluation of the GT uncertainty during AI model development [67]. The reliance on annotations from a single grader or the absence of an inter-rater variability assessment can introduce biases into the training data, potentially leading AI models to learn subjective patterns. In an everyday routine, this could result in inconsistent performance across different scenarios, particularly when models encounter variations in the annotation styles of different clinicians.

Another important limitation was the low rate of external validation (only 16.7% of studies) [41,50,52,56], underlining the need for additional validation using unseen conditions. In real-world settings, segmentation tasks often encounter several challenges, like imaging artefacts, interfering noise, poor contrast, and GA lesions or general anatomic variability, raising the bar for algorithmic performance under difficult-to-segment conditions while maintaining reliable and accurate results [23]. AI models without external validation can be unreliable and clinically meaningless. We strongly encourage AI researchers to incorporate external testing into their work to overcome this significant limitation and produce robust and generalizable algorithms before exploiting their great clinical potential.

We also noted the limited use of XAI techniques, with only five studies [40,45,46,48,50] incorporating such methods. The overall lack of transparency in the AI literature on GA segmentation underscores the need for more XAI models in ophthalmology that, apart from performing impressively well, also demonstrate confidence, trustworthiness, and credibility. Overcoming this challenge is essential before these algorithms can be integrated into critical decision-making roles in clinical care [68]. Additionally, ambiguity in the XAI terminology, with no clear and consistent definitions across domains, makes the implementation and evaluation of such technologies challenging [27].

Furthermore, a lack of coherently reported evaluation metrics was also noted, with the studies using variable indices for model performance assessment. Four studies omitted the DSC or F1 [38,47,49,54], while three of them [38,47,49] used the overlap ratio for model–grader agreement evaluation. One study [54] showed inadequate evaluation reporting, mentioning only the sensitivity, specificity, false positives, and correct GA border identification, which is a non-standardized metric. The accuracy, AAD, precision, and AUC indices were insufficiently reported, potentially limiting the comparability of the segmentation studies. We strongly recommend the use of diverse evaluation metrics, as incorporating a range of measures offers a more comprehensive assessment of model performance, highlights the strengths and weaknesses, and promotes trust in AI-driven segmentation.

Another common limitation was the decline in model performance when evaluating small, irregular, extramacular, low-contrast, poorly illuminated, and blurry margin lesions, as well as in cases with drusen co-existence or interfering retinal blood vessels. These cases are common in routine practice and stress the importance of avoiding inappropriate exclusions or selecting “textbook examples” of GA for model development. Such models may struggle with borderline cases or overfit to ideal features. Again, we highlight the need for large, diverse GA datasets, as well as proper model training, validation, and external testing to overcome the aforementioned limitations [69].

Furthermore, our extensive CLAIM assessment results revealed inconsistencies among the studies. Future studies should adopt high reporting standards to enhance the reliability and reproducibility of their models. According to a 2019 systematic review of ML applications in medical imaging, only 2.4% of studies reported how they determined the sample size used [70], a limitation also observed in our study cohort. While the original review did not explore the reasons behind this omission, it underscores the need for standardized guidance on sample size justification in AI research. Clearly defining and reporting the sample size—particularly for the training, validation, and testing subsets—can support methodological rigor, and improve the efficiency and generalizability of AI models.

We also observed that only a small number of the studies incorporated patient demographic data or flowcharts detailing the inclusion/exclusion processes. While this information may seem redundant for training AI models, it contextualizes a dataset and exposes the underrepresented subpopulations. GA lesion variability among different ethnic groups has been documented [71]. Τherefore, epidemiological patient data are valuable for fair and unbiased medical AI models. At the same time, participant flow diagrams clarify the data selection process, help prevent data leakage, and contribute to overall transparency.

Another important aspect is the disclosure of patient data anonymization strategies, which were not comprehensively reported across the studies. The recent implementation of the AI Act [72] marks a significant effort towards regulating AI research while prioritizing personal safety and privacy. Especially in the case of healthcare-related models, the AI Act seeks to protect the potential leakage of patient medical records and, in conjunction with the General Data Protection Regulation (GDPR), obliges AI models to safeguard the fundamental rights of individuals and preserve their anonymity [73]. Other established regulatory efforts include the Health Insurance Portability and Accountability Act (HIPAA) and the European Health Data Space (EHDS), which also aim to preserve the security of patient records and promote responsible and transparent data handling [74]. AI models will henceforth be obliged to meet the requirements of these regulatory frameworks and employ strong de-identification strategies that protect sensitive patient data and other biometric information, while sharing the relevant details for enhanced reporting quality and transparency [33,75].

Equally important, the QUADAS-2 quality assessment indicated that AI developers should acknowledge that redundant exclusions may lower models’ real-world translation potential and present detailed model development information, especially any important standardization methods and performance cutoffs.

While the limitations of the included studies are evident, it is also important to acknowledge the limitations of our own review. This work presents a balanced report of the research efforts undertaken thus far on AI applications for GA segmentation, detailing 24 prominent studies and showcasing the impressive performance results of our meta-analysis while also charting the areas requiring further investigation or increased reporting attention. In terms of the quantitative synthesis, although moderate heterogeneity was observed across these studies, likely due to differences in the AI models and imaging sources, the random-effects model accounts for such variability. Future meta-analyses could benefit from subgroup analyses (e.g., by imaging modality or algorithm type) once a larger pool of standardized datasets and models becomes available. Regarding the broader review, we did not interrogate AI-targeted databases, such as IEEE Xplore or ACM Digital Library, potentially limiting the comprehensiveness of this review. However, our primary focus was on medical imaging for a specialized ophthalmic disease, GA, which was expected to be broadly referenced in the databases selected for this review. Furthermore, our manual search attempts did not yield additional relevant results. It is also important to acknowledge the possibility of publication bias, as studies reporting lower segmentation performance (e.g., lower DSC values) may be less likely to be published. This could lead to an overestimation of the pooled performance, despite our comprehensive and systematic search strategy. Another potential source of bias in our findings could be the lack of a clear GA definition and the concurrent introduction of the CAM* terminology. We chose not to focus solely on the retinal pigment epithelium and outer retinal atrophy (RORA), which—although precisely defined and structured as an imaging term—is still not widely utilized in everyday clinical practice, where GA remains the most frequent diagnosis. Our systematic review aimed to provide a concise summary of model performance under both GA and RORA umbrella terms, ultimately presenting findings with actual clinical value. We also recognize that the concept of segmentation in medical imaging can sometimes be mislabeled as “quantification” or “detection”, without a clear explanation of a model’s output and its agreement with our definition of GA segmentation. For this reason, we only included studies in which it was clearly evident that segmentation was correctly addressed. Lastly, at the time this review was conducted, no AI-specific quality assessment tools had been released and the tools we employed did not evaluate the integral parts of AI methodology, such as explainability or preprocessing bias.

Despite these limitations, the findings of our review highlight several important opportunities for the future advancement of AI-based GA segmentation. These approaches have the potential to deliver significant public health benefits by reducing clinical workloads, lowering the doctor-to-patient ratio, enhancing initial screening in remote areas, improving monitoring accuracy, and minimizing the risk of misdiagnoses or missed lesions. Additionally, AI models can optimize recruitment procedures for clinical trials with strict inclusion criteria and may assist in identifying novel biomarkers or endpoints, supporting the work of both clinicians and researchers.

To fully realize these benefits, several areas should be refined in future research. The development of GA-specific models trained on large, diverse, multicenter datasets is essential to ensure robust performance across real-world clinical scenarios. These datasets should encompass variability in image quality, GA phenotypes, and sociodemographic characteristics, and ideally be acquired through multimodal imaging platforms in accordance with local or international data protection regulations. External testing and clinical validation under unseen conditions are also critical for demonstrating the reliability and generalizability of AI models in clinical practice. Similarly, the challenge of manual labeling must be addressed through strategies, such as the creation of standardized annotation protocols, semi-automated labeling tools, or collaborative labeling efforts, which could improve the ground-truth quality while minimizing the time, cost, and labor.

While the primary focus of this review was not on predictive modeling or future progression algorithms, we recognize that the clinical value of segmentation models extends beyond static assessments. In particular, their utility in longitudinal studies—such as tracking GA progression and estimating annual growth rates—relies not only on segmentation accuracy but also on temporal consistency across serial scans. Advancing research in this area will be crucial for translating segmentation models into tools that can support long-term disease monitoring and structural endpoint assessments in drug trials.

Ongoing AI research is advancing at a high rate, with algorithms constantly improving and our understanding of their behavior steadily expanding. In addition to the current deep learning techniques, next-generation architectures, such as U-Net++ [76], V-Net [77], and diffusion models [78], are increasingly being explored and may lead to more refined and clinically applicable segmentation solutions. Moreover, explainable AI is becoming more common, helping make AI models easier to understand and more transparent, important features for gaining trust in clinical settings. The future of AI-based GA segmentation is highly promising, and the trajectory suggests it will significantly contribute to accurate, trustworthy, and high-precision ophthalmic care and research.

## 5. Conclusions

GA is a significant cause of compromised vision, and accurate lesion segmentation remains a complex and detailed process in both clinical and research contexts. AI offers the potential to automate, optimize, and scale this task, ultimately improving patient care. Our findings demonstrate the remarkable predictive capabilities of AI models for GA segmentation, highlighting the promising opportunities resulting from ongoing algorithmic advances. However, high performance alone is insufficient for clinical integration. Key challenges—including the need for improved explainability, robust external validation, and access to large, diverse datasets—must be addressed to ensure greater transparency, reliability, and generalizability. This review provides a comprehensive overview of the current capabilities and limitations of AI-based GA segmentation and offers a valuable foundation for future research. Addressing the current limitations will be essential for unlocking the full potential of AI in ophthalmology and facilitating its meaningful adoption in clinical care.

## Figures and Tables

**Table 1:** PICOS criteria for study selection.

PICOS	Description
Population	Adult patients with geographic atrophy due to advanced dry age-related macular degeneration.
Intervention	Artificial intelligence algorithms for the segmentation of geographic atrophy. Various AI tools trained to automatically delineate geographic atrophy lesions using different retinal imaging modalities (CFP, OCT, FAF, etc.).
Comparator	Alternative segmentation methods: manual segmentation performed by human experts, validated semi-automated segmentation techniques, or other software-assisted methods.
Outcomes	Accuracy and efficacy of artificial intelligence techniques for segmenting geographic atrophy as measured through performance metrics (sensitivity, specificity, AUC, F1 score, IoU, DSC, etc.).
Study Design	Studies included observational studies, randomized clinical trials, and registry/database studies.
Additional Details	Language: English. Time frame: from inception to 23 March 2024. Location: worldwide.

AUC (area under the curve); CFP (color fundus photography); DSC (Dice similarity coefficient); FAF (fundus autofluorescence); IoU (intersection over union); OCT (optical coherence tomography).

**Table 2:** Eligibility criteria for study selection.

Inclusion Criteria	Exclusion Criteria
Observational studies, randomized clinical trials, registry/database studies.	Systematic reviews, meta-analyses, narrative reviews, scoping reviews, opinion pieces, surveys, editorials, commentary letters, case reports, book chapters, conference abstracts, proceedings, or presentations.
Studies using artificial intelligence techniques.	Studies using traditional image processing techniques.
Studies including eyes of adult patients.	Studies including pediatric patients, geographic atrophy due to neovascular age-related macular degeneration, or non-human studies.
Studies addressing geographic atrophy lesion segmentation with the use of artificial intelligence segmentation techniques.	Studies addressing solely geographic atrophy diagnosis, classification, progression, future prediction, treatment response, area quantification, or retinal layer-by-layer segmentation.
Studies reporting at least one of the following metrics: accuracy, sensitivity/recall, specificity, precision/PPV, F1 score, AUC/AUROC, AUPR, IoU/OR, DSC/SDC, or AAD.	Studies without clear documentation of the purpose, dataset, data sources, patient distribution, methods, results, or conclusions.
Articles published in English language.	Ineligible population and ineligible outcome.
	Articles with full text unavailable.

AAD (absolute area difference); AUC (area under the curve); AUPR (area under the precision-recall curve); AUROC (area under the receiver operating characteristic curve); DSC (Dice similarity coefficient); IoU (intersection over union); OR (overlap ratio); PPV (positive predictive value); SDC (symmetric Dice coefficient).

**Table 3:** Categories of extracted data.

Category	Description
Study information	Author Year Country (affiliations of all contributing authors)
Dataset and annotation methods	*Diseases* (all diseases included in the datasets) CAM* (adherence to CAM* consensus definitions of geographic atrophy) [6] *Dataset* (datasets used for model development) *Imaging modality* (main imaging devices used by the model, specifying those used for model development and those used for ground truth establishment, including manufacturer details when reported) *Total patients/eyes* (number of patients and eyes used for model development) *Total sample size* (size and type of data contained in the datasets) *GA sample size (% of dataset)* (size of geographic atrophy segmentation subset and its percentage relative to the entire development dataset) *Ground truth* (strategy used for reference standard establishment)
AI model characteristics and validation strategy	*AI type* (deep learning or machine learning) *AI algorithm* (basic model architecture) *Segmentation technique* (brief description of the geographic atrophy segmentation method) *XAI* (use of explainable AI techniques and the approach applied) *Internal validation method* (approach used during model training to evaluate performance and prevent overfitting, such as k-fold cross-validation, hold-out validation, etc.) *Training set/validation set/internal test set* (size and type of data partitioning for each geographic atrophy segmentation model) *External test set* (use of an unseen independent dataset, including dataset name and size when reported)
Model performance evaluation	*Performance metrics for internal and external testing* (reporting of accuracy, sensitivity/recall, specificity, precision/PPV, F1 score, AUC/AUROC, AUPR, IoU/OR, DSC/SDC, and AAD for geographic atrophy segmentation)

AAD (absolute area difference); AUC (area under the curve); AUPR (area under the precision-recall curve); AUROC (area under the receiver operating characteristic curve); CAM* (Classification of Atrophy Meetings); DSC (Dice similarity coefficient); GA (geographic atrophy); IoU (intersection over union); OR (overlap ratio); PPV (positive predictive value); SDC (symmetric Dice coefficient); XAI (explainable artificial intelligence).

**Table 4:** Study characteristics of datasets and annotation methods.

Author (Year)	Country	Diseases	CAM*	Dataset	Imaging Modality	Total pts/Eyes	Total Sample Size	GA Sample Size (% of Dataset)	Ground Truth
Arslan (2021) [34]	Australia	GA secondary to dry AMD	No	Institutional dataset (Centre for Eye Research Australia) and private dataset	FAF (Heidelberg Spectralis)	51/99	702 FAF images	702 FAF images (100% of dataset)	Manual annotation of FAF by principal grader, additional grading of subset by senior grader for GT evaluation, and RegionFinder software outputs for comparison.
Chu (2022) [35]	USA	GA and Healthy	Yes	Institutional dataset (part of the University of Miami)	SS-OCT (PLEX Elite 9000 Carl Zeiss)	115/140	184 OCT scans	124 OCT scans (67.3% of dataset)	Manual annotation by 2 independent graders reaching consensus on the en face subRPE OCT images (with senior adjudicator).
Derradji (2021) [36]	Switzerland	Atrophic AMD with RORA and Healthy	Yes	Institutional dataset (JG Eye Hospital Medical Retinal Department)	SD-OCT (Heidelberg Spectralis)	57/62	62 OCT volumes (3595 B-scans)	2085 B-scans with RORA (58% of dataset)	Manual annotation by 2 expert graders (senior and junior).
Domalpally (2024) [50]	USA	GA	No	AREDS2 subset	FAF (Heidelberg Spectralis HRA)	271/362	601 FAF images	601 FAF images (100% of dataset)	Manual GA demarcation by certified graders.
Feeny (2015) [51]	USA Thailand	GA	No	AREDS dbGAP subset	CFP	NR/55	143 CFP images	143 CFP images (100% of dataset)	Manual delineation by 1 retina specialist and review/correction by retina specialist team.
Hassan (2021) [37]	China, UAE	11 CRBMs from a pool of datasets containing AMD, ME, CSCR, MH, DR, CNV, and Healthy	No	7 OCT datasets spread over 4 DBs (Isfahan University of Medical Sciences, Duke University, University of Waterloo, and Guangzhou Medical University)	OCT (Spectralis, Cirrus, Topcon, and Bioptigen)	6953/NR	7000 OCT scans	500 OCT scans (7.1% of dataset)	Manual pixel-wise annotations of 11 biomarkers by 3 retina specialists (1 grader per scan).
Hu (2015) [38]	USA	GA	No	Institutional FAF dataset (University of Southern California Retina Clinics)	FAF (Heidelberg cSLO Spectralis HRA + OCT)	16/16	16 FAF images	16 FAF images (100% of dataset)	Manual delineation by certified grader.
Ji (2018) [47]	China, USA	Advanced non- exudative AMD with GA	No	2 datasets previously described and used [59]	SD-OCT (Cirrus Carl Zeiss) FAF (for GT annotations)	62/66	51 SD-OCT cube scans (DS1); 54 SD-OCT cube scans (DS2) and corresponding FAF images	105 SD-OCT cube scans (100% of DS1 and DS2)	DS1: manual outline average of 2 independent experts from 2 sessions, based on B-scans. DS2: manual outline based on FAF images and manual registering to projection images.
Kalra (2022) [39]	USA	Nonexudative AMD with or without GA (including iRORA and cRORA)	Yes	Institutional OCT dataset (Cole Eye Institute of the Cleveland Clinic)	SD-OCT (Heidelberg Spectralis HRA + OCT and Cirrus HD-OCT Zeiss)	341/NR	100.266 SD-OCT B-scans and 900 en face SD-OCT images	NR	B-scan model: Retinal layer segmentation masks by previously validated ML model, corrected by expert reader, reviewed by senior expert, and adjudicated by analysis director. Binary GA masks in areas of outer layer attenuation overlap. En face model: generated from assembled B-scan masks.
Liefers (2020) [52]	The Netherlands, Australia, Singapore, Switzerland	AMD with GA	No	BMES and RS cohorts I, II, and III	CFI (Zeiss, Canon CF-60 Dsi, Topcon TRV-50VT/TRC 50EX, and Sony DXC-950P)	175/238	409 CFI images	409 CFI images (100% of dataset)	Manual delineation by 4 expert graders for BMES and at least 2 expert graders for RS using in-house software and final consensus grading. Consultation with additional multimodal data when available.
Liu (2023) [40]	China, USA	GA, Healthy, and other CRBMs (IRF/DME, DRUSEN, CNV, PED, HF, and SRF)	No	Private retinal biomarker dataset from Wuhan Aier Eye Hospital and Kermany’s public dataset	OCT Triton DRI for DS1, NR for DS2)	NR/NR	30.850 B-scans	700 B-scans (2.27% of dataset)	Previously published manual annotations for IRF/DRUSEN and ophthalmologist annotations for CNV. Annotation methods for GA are not reported.
Ma (2020) [48]	China, USA	Advanced non- exudative AMD with GA	No	2 datasets previously described and used [59]	SD-OCT (Cirrus Carl Zeiss) FAF (for GT annotations)	62/66	105 SD-OCT cube scans	105 SD-OCT cube scans (100% of dataset)	DS1: manual outline by 2 independent experts from 2 sessions based on B-scans. DS2: manual outline by 1 expert based on FAF images and mapping to projection image.
Mai (2023) [41]	Austria	GA	No	Institutional dataset (Medical University of Vienna)	SD-OCT (Heidelberg Spectralis) FAF (for GT annotations)	100/184	967 OCT volumes	967 OCT volumes (100% of dataset)	Manual annotation of FAF by 2 trained graders using OCTAVO software, with automatic NIR registration and final 2D en face OCT annotations.
Morano (2024) [42]	Austria	GA	No	Institutional dataset (Medical University of Vienna)	OCT, SLO, and FAF (Heidelberg Spectralis)	100/184	967 OCT volumes, including OCT B-Scans, SLO, and FAF images	967 OCT volumes, including OCT B-Scans, SLO, and FAF images (100% of dataset)	En face masks annotated by 1 retinal expert on FAF, additional annotations on 35 OCT samples by retinal experts, with automatic co-registration of OCT-SLO and FAF-SLO.
Pfau (2020) [53]	USA, Germany	GA and Healthy	No	Directional spread in GA 2 study dataset	FAF, IR, and SD-OCT (Spectralis HRA + OCT2)	182/251	UC	UC	B-scan and en face manual segmentation by 2 readers on test set (3 B-scans per patient).
Pramil (2023) [57]	USA, Australia	GA, Early AMD, Intermediate AMD, and Healthy	No	Study OCT dataset (“SWAGGER” cohort) and institutional dataset from New England Eye Center (Tufts Medical Center)	SS-OCT (PLEX Elite 9000 Carl Zeiss)	138/198	351 OCT scans	273 OCT scans (77.7% of dataset)	Plex Elite Review software layer segmentation, then manual annotations on generated en face images by 1 expert grader for development set and 2 expert graders during 2 separate sessions for test set (ophthalmology research fellows), with grader training and annotation verification by retina specialist.
Ramsey (2014) [54]	USA	GA	No	Fenretinide study subset	FAF and CFP	10/NR	26 FAF-CFP image pairs	26 FAF-CFP image pairs (100% of dataset)	Manual delineation by expert grader.
Royer (2021) [43]	France	GA	No	Institutional dataset (Clinical Imaging Center, Quinze-Vingts Hospital)	cSLO	13/NR	328 cSLO images	328 cSLO images (100% of dataset)	GA delineation by ophthalmologists.
Spaide (2023) [55]	USA, Germany	GA	No	Proxima A and Proxima B study datasets	FAF and NIR (Spectralis cSLO)	337/337	1437 FAF-NIR image pairs	1437 FAF-NIR image pairs (100% of dataset)	Semi-automatic delineation on FAF by trained graders using RegionFinder software. DS1 later assessed by 2 junior graders with senior adjudicator. DS2 later assessed by junior and senior grader with optional second senior grader involvement. Total of 384 images used for GT.
Szeskin (2021) [44]	Israel	cRORA and Macular atrophy (cRORA, iRORA, cORA, and iORA)	Yes	2 institutional datasets (Hadassah University Medical Center)	OCT and IR (Heidelberg Spectralis)	34/NR	106 OCT scans + IR (5.207 slices) (DS1); 19 OCT scans + IR (829 slices) (DS2)	2952 slices (56.70% of DS1); 829 slices (100% of DS2)	DS1: multistep manual annotation of OCT by 2 technical co-authors and 2 ophthalmologists (of which one was senior reviewer). DS2: Manual delineation on IR image by medical student and projection onto OCT. Both DSs used in-house OCT-E GUI software.
Wang (2022) [45]	USA	AMD GA and Stargardt atrophy	No	In-house datasets	FAF (Spectralis HRA + OCT)	217/296	180 FAF images (AMD GA DS); 412 FAF images (Stargardt DS)	90 FAF images (50% of AMD GA dataset) (15.2% of both datasets)	Manual delineation/labeling of FAF by certified grader using RegionFinder software and reviewed by senior grader with senior adjudicator.
Wu (2019) [46]	China, USA, South Korea	Advanced non- neovascular AMD with GA	No	In-house dataset	SD-OCT (Cirrus Carl Zeiss) FAF (Heidelberg Spectralis)	56/NR	56 SD-OCT volumes	56 SD-OCT volumes (100% of dataset)	Manual segmentation of FAF images and manual registration on en face OCT by fellowship-trained retinal specialist.
Xu (2019) [49]	China, USA	GA	No	2 datasets previously described and used [59]	SD-OCT (Carl Zeiss)	64/NR	55 SD-OCT cubes (DS1); 56 SD-OCT cubes with corresponding FAF images (DS2)	111 SD-OCT cubes (100% of DS1 and DS2)	DS1: manual outline of OCT cubes by 2 ophthalmologists during 2 sessions. DS2: manual outline based on FAF image by ophthalmologist and registered to projection image.
Zhang (2021) [56]	UK, the Netherlands, Switzerland	GA (including cRORA and iRORA)	Yes	FILLY dataset	OCT (Heidelberg Spectralis OCT + HRA)	200/399	984 OCT volumes (5049 B-scans)	984 OCT volumes (100% of dataset)	Manual annotation of 5 B-scans per OCT volume by 3 expert graders.

AMD (age-related macular degeneration); AREDS (age-related eye disease study); BMES (Blue Mountains eye study); CAM* (Classification of Atrophy Meetings); CFI (color fundus image); CFP (color fundus photography); CNV (choroidal neovascularization); cORA (complete outer retinal atrophy); cRORA (complete retinal pigment epithelium and outer retinal atrophy); CRBM (chorioretinal biomarker); CSCR (central serous chorioretinopathy); cSLO (confocal scanning laser ophthalmoscopy); DB (database); DME (diabetic macular edema); DR (diabetic retinopathy); DS (dataset); FAF (fundus autofluorescence); GA (geographic atrophy); GT (ground truth); GUI (graphical user interface); HD-OCT (high-definition optical coherence tomography); HF (hyperreflective foci); iORA (incomplete outer retinal atrophy); IR (infrared reflectance); IRF (intraretinal fluid); iRORA (incomplete retinal pigment epithelium and outer retinal atrophy); ME (macular edema); MH (macular hole); NIR (near-infrared reflectance); NR (not reported); OCT (optical coherence tomography); PED (pigment epithelial detachment); RORA (retinal pigment epithelium and outer retinal atrophy); RS (Rotterdam study); SD-OCT (spectral-domain optical coherence tomography); SLO (scanning laser ophthalmoscopy); SRF (subretinal fluid); SS-OCT (swept-source optical coherence tomography); subRPE (sub-retinal pigment epithelium); UC (unclear).

**Table 5:** Segmentation methods and validation approaches of selected studies.

Author (Year)	Segmentation Technique	Internal Validation Method	Training Set	Validation Set	Internal Test Set	External Test Set
Arslan (2021) [34]	Pixel prediction and classification—binary classification problem.	5-fold cross-validation	5 sets of 140 or 142 FAF images each	5 sets of 140 or 142 FAF images each	5 sets of 140 or 142 FAF images each	NR
Chu (2022) [35]	2 models (trained on OAC false-color en face images or OCT subRPE en face images).	train–test split	80% of all eyes (89 eyes—133 OCT scans)	20% of training set (23 eyes—23 OCT scans)	20% of all eyes (28 eyes—28 OCT scans)	NR
Derradji (2021) [36]	CNN trained using single 2D B-scan as input and producing corresponding 2D RORA probability mask as output.	5-fold cross-validation on the merged training and validation sets	2301 OCT B-scans	256 OCT B-scans	1038 OCT B-scans	NR
Domalpally (2024) [50]	Weakly labeled model for GA measurement and strongly labeled model for GA measurement and pixel-level segmentation.	5-fold cross-validation	80% of dataset (~481 FAF images) for each iteration	20% of dataset (~120 FAF images) for each iteration	NR	156 FAF images from GSK BAM114341 study dataset
Feeny (2015) [51]	52 features computed per pixel for binary classification (GA vs. not-GA).	Leave-one-out cross-validation	142 CFP images for each iteration	NR	1 CFP image for each iteration	NR
Hassan (2021) [37]	Asymmetric encoder–decoder structure for joint segmentation and quantification of 11 CRBMs with preprocessing, feature map utilization, and postprocessing stages.	Hold-out validation	4200 OCT B-scans (352 for GA) from University of Waterloo DB and Guangzhou Medical University DB	1400 OCT B-scans in total (75 for GA)	1400 OCT B-scans in total (73 for GA) from Isfahan University of Medical Sciences DB and Duke University DB	NR
Hu (2015) [38]	Supervised pixel classification employing image texture features.	8-fold cross-validation	8 rotating FAF image sets	NR	8 rotating FAF image sets	NR
Ji (2018) [47]	Deep voting model with 5 layers. A-scans labeled with 1024 features fed into the network and a soft-max classifier determined pixel-level labels. 10 trained models. No retinal layer segmentation.	Hold-out validation	10.000 GA-positive OCT A-scans and 10.000 GA-negative OCT A-scans for each dataset (~5% and ~9% of total data)	NR	NR	NR
Kalra (2022) [39]	2 models (B-scan and en face) for binary detection of GA presence and pixel-wise lesion segmentation.	Hold-out validation	80% of total patients	10% of total patients	Unseen 10% of patients	NR
Liefers (2020) [52]	Ensemble of 20 models obtained during 5-fold cross-validation with average of pixel-wise predictions used to form a single binary image.	5-fold cross-validation	~254 CFI images for each model (60% of data)	NR	NR	50 random CFIs from AREDS subset
Liu (2023) [40]	3 stages: a supervised, contrastive learning-based pre-training; a fine-tuning module combining two loss functions; and a knowledge distillation-based teacher–student network for anomaly localization.	Hold-out validation	500 GA OCT B-scans	100 GA OCT B-scans	100 GA OCT B-scans	NR
Ma (2020) [48]	Stage 1: B-scan denoising, RPE segmentation, flattening. Stage 2: B-scans input into weakly supervised network to generate attention maps. Stage 3: Segmentation masks by graph-based algorithm using positive/negative seeds.	5-fold cross-validation	NR	NR	NR	NR
Mai (2023) [41]	Patch-based training, 3D to 2D image segmentation.	5-fold cross-validation	~695 OCT volumes (90% of development set)	~77 OCT volumes (10% of development set)	~193 OCT volumes	226 OCT volumes from FILLY dataset
Morano (2024) [42]	Multimodal Late Fusion and Multiscale Fusion approaches with 2 branches to extract and project features from different modalities and dimensions (3D OCT and 2D FAF or SLO images) onto a common feature subspace, enabling their joint use for segmentation tasks.	Hold-out validation	~580 OCT volumes (B-scans, SLO, and FAF) (60% of DS)	~97 OCT volumes (B-scans, SLO, and FAF) (fixed 10% of DS)	~290 OCT volumes (B-scans, SLO, and FAF) (fixed 30% of DS)	NR
Pfau (2020) [53]	1st CNN for 6-layer retinal segmentation, and multimodal input stacking into 2nd CNN for en face GA segmentation.	Hold-out validation	135 eyes for en face GA segmentation	45 eyes for en face GA segmentation	75 OCT B-scans of 25 patients with GA	NR
Pramil (2023) [57]	Encoding of OCT-derived GA features onto a pseudocolor image using RGB channels for RPE loss, hypertransmission, and retinal thinning, respectively.	5-fold cross-validation	126 OCT scans	~25 OCT scans (~20% of training set)	225 OCT scans	NR
Ramsey (2014) [54]	Image registration, digital vessel subtraction, user-defined ROI, soft FCM segmentation, user selection of relevant topographies, and final GA quantification.	NR	NR	NR	NR	NR
Royer (2021) [43]	Unsupervised fully convolutional autoencoder trained on 2 loss functions (reconstruction error and soft N-cut loss) and 3 classes to segment GA by maximizing pixel intensity and spatial dissimilarity.	8 different random combinations of 12 series for training and 6 for testing	NR	NR	NR	NR
Spaide (2023) [55]	Single encoder–decoder architecture for pixel prediction and classification (UNet), two encoder branches to encode FAF and NIR images separately, and one joint decoder to decode the embeddings (Ynet).	Hold-out validation	748 FAF-NIR image pairs from DS2	192 FAF-NIR image pairs from DS2	497 FAF-NIR image pairs from DS1	NR
Szeskin (2021) [44]	CNN that classified light-scattering patterns in 2D and 3D columns of vertical pixel-wide vectors (A-scans) on atrophic OCT B-scans, utilizing the BCE and F1 loss functions without layer segmentation.	4-fold cross-validation	93 OCT scans for cRORA; 10 OCT scans for macular atrophy for training and cross-validation	93 OCT scans for cRORA; 10 OCT scans for macular atrophy for training and cross-validation	12 OCT scans for cRORA; 9 OCT scans for macular atrophy for testing	NR
Wang (2022) [45]	Integrated soft-labeled self-attended deep CNN system and binary pixel classification producing feature maps.	8-fold cross-validation	70 AMD FAF images (in each fold)	10 fixed AMD FAF images	10 AMD FAF images (in each fold)	NR
Wu (2019) [46]	FAF image synthesis from generated en face OCT by GA RA-CGAN; image fusion net and segmentation net refined with SFCM.	Leave-four-out cross-validation	NR	NR	NR	NR
Xu (2019) [49]	Offline learning phase to capture common features from training samples and self-learning phase to identify discriminative features and reduce FPs. Fusion of both outputs for final segmentation.	Independent dataset validation	Random 100.000 axial data with GA and 100.000 without GA for each DS	NR	111 3D OCT cubes in total from both DSs	NR
Zhang (2021) [56]	2 approaches: direct GA segmentation or individual segmentation of overlapping features (RPE loss, photoreceptor degeneration, and hypertransmission).	Hold-out validation	582 OCT volumes (3024 B-scans) (60% of dataset)	191 OCT volumes (958 B-scans) (20% of dataset)	211 OCT volumes (1067 B-scans) (20% of dataset)	192 OCT volumes (884 B-scans) from Moorfields Eye Hospital institutional dataset

AMD (age-related macular degeneration); AREDS (age-related eye disease study); BCE (binary cross-entropy); CFI (color fundus image); CFP (color fundus photography); CNN (convolutional neural network); cRORA (complete retinal pigment epithelium and outer retinal atrophy); CRBM (chorioretinal biomarker); DB (database); DS (dataset); FAF (fundus autofluorescence); FCM (fuzzy c-means clustering); FP (false positive); GA (geographic atrophy); NIR (near-infrared reflectance); NR (not reported); OAC (optical attenuation coefficient); OCT (optical coherence tomography); RA-CGAN (region-aware conditional generative adversarial network); RGB (red–green–blue); ROI (region of interest); RORA; (retinal pigment epithelium and outer retinal atrophy); RPE (retinal pigment epithelium); SFCM (spatial fuzzy c-means clustering); SLO (scanning laser ophthalmoscopy); subRPE (sub-retinal pigment epithelium).

**Table 6:** Performance evaluation, explainability, and architecture of AI models.

Author (Year)	AI Type	AI Algorithm	XAI	Performance Metrics (Internal Testing)	Performance Metrics (External Testing)
Arslan (2021) [34]	DL	U-Net	NR	Accuracy: 0.9774; Sensitivity: 0.9903; Specificity: 0.7498; Precision: 0.9837; DSC: 0.9780	NR
Chu (2022) [35]	DL	U-Net	NR	**OAC composite model:** GA Identification Sensitivity: 100% GA Identification Specificity: 100%; DSC: 0.940 **OCT en face subRPE model**: GA Identification Sensitivity: 100% GA Identification Specificity: 100%; DSC: 0.889	NR
Derradji (2021) [36]	DL	U-Net style network with EfficientNet-b3 architecture	NR	**Model with prior layer:** Sensitivity (grader 1): 0.850; Sensitivity (grader 2): 0.915 Precision (grader 1): 0.928; Precision (grader 2): 0.799 DSC (grader 1): 0.881; DSC (grader 2): 0.844 **Model without prior layer:** Sensitivity (grader 1): 0.765; Sensitivity (grader 2): 0.845 Precision (grader 1): 0.955; Precision (grader 2): 0.845 DSC (grader 1): 0.841; DSC (grader 2): 0.831	NR
Domalpally (2024) [50]	DL	EfficientNet-B5 (weakly labeled model) Feature Pyramid Network with EfficientNet-B5 encoder (strongly labeled model)	Saliency maps	**Strongly labeled model:** DSC: 0.885	**Strongly labeled model:** DSC: 0.918
Feeny (2015) [51]	ML	Random forest classifier	NR	Sensitivity: 0.65; Specificity: 0.99; PPV: 0.82; DSC: 0.68	NR
Hassan (2021) [37]	DL	RASP-Net	NR	**GA detection:** Sensitivity: 0.904; Specificity: 0.930; Precision: 0.853 **GA segmentation:** IoU: 0.606; DSC: 0.755	NR
Hu (2015) [38]	ML	k-NN	NR	Accuracy: 0.94; Sensitivity: 0.87; Specificity: 0.96; PPV: 0.80; OR: 0.72	NR
Ji (2018) [47]	DL	Deep voting model (sparse autoencoders)	NR	**DS1:** AAD (mm^2^): 0.67; AAD (%): 11.49; OR: 0.8694 **DS2: ** AAD (mm^2^): 0.34; AAD (%): 8.30; OR: 0.8166	NR
Kalra (2022) [39]	DL	U-Net	NR	**B-scan model:** Detection Accuracy: 0.91; Pixel-wise Accuracy: 0.94 Detection Sensitivity: 0.86; Pixel-wise Sensitivity: 0.90 Detection Specificity: 0.94; Pixel-wise Specificity: 0.90 Detection f-score: 0.87; Pixel-wise f-score: 0.71 **En face model:** Detection Accuracy: 0.82; Pixel-wise Accuracy: 0.96 Detection Sensitivity: 0.97; Pixel-wise Sensitivity: 0.95 Detection Specificity: 0.42; Pixel-wise Specificity: 0.93 Detection f-score: 0.88; Pixel-wise f-score: 0.82	NR
Liefers (2020) [52]	DL	Deep encoder–decoder structure with residual blocks and shortcut connections	NR	DSC: 0.72	DSC: 0.66 (total cases); DSC: 0.71 (pure GA cases)
Liu (2023) [40]	DL	TSSK-Net with ResNet-18 backbone	CAM-based techniques (Grad-CAM, LayerCAM, and MS-CAM) and knowledge distillation-based anomaly localization (heatmaps)	**GA Segmentation:** AUC: 0.9704; DSC: 0.4504	NR
Ma (2020) [48]	DL	VGG16 backbone	Saliency maps (MS-CAM)	**DS1:** AAD (mm^2^): 0.63; AAD (%): 12.87; AUC: 0.9417; OR: 0.8430; DSC: 0.9121 **DS2:** AAD (mm^2^): 0.78; AAD (%): 14.99; AUC: 0.9326; OR: 0.7441; DSC: 0.847	NR
Mai (2023) [41]	DL	DL model based on U-Net architecture	NR	Recall: 0.87; Precision: 0.87; DSC (mean): 0.86; DSC (median): 0.90	Recall: 0.93; Precision: 0.90; DSC: 0.91; DSC (mean): 0.91; DSC (median): 0.93
Morano (2024) [42]	DL	FCNN	NR	**100% of training data** **Multiscale OCT + FAF:** AUROC: 0.996; AUPR: 0.9895; DSC: 0.9019 **Multiscale OCT + SLO:** AUROC: 0.9939; AUPR: 0.984; DSC: 0.8915 **Late OCT + FAF:** AUROC: 0.9948; AUPR: 0.9859; DSC: 0.8991 **Late OCT + SLO:** AUROC: 0.9933; AUPR: 0.9822; DSC: 0.8908	NR
Pfau (2020) [53]	DL	Deeplabv3 model with ResNet-50 backbone	NR	DSC: 0.96	NR
Pramil (2023) [57]	DL	U-Net	NR	**GA detection:** Sensitivity: 0.95; Specificity: 0.91 **GA segmentation:** SDC (grader 1): 0.92; SDC (grader 2): 0.91; SDC (after BM correction): 0.97	NR
Ramsey (2014) [54]	ML	FCM	NR	Sensitivity (FAF): 0.94; Sensitivity (CFP): 0.47 Specificity (FAF): 0.98; Specificity (CFP): 0.98	NR
Royer (2021) [43]	DL	W-net (autoencoder)	NR	Sensitivity: 0.85; Precision: 0.90; F1 score: 0.87	NR
Spaide (2023) [55]	DL	Y-Net and U-Net	NR	DSC: 0.90–0.92	NR
Szeskin (2021) [44]	DL	Custom column-based CNN	NR	**cRORA**: Mean Detection Recall: 0.67; Mean Detection Precision: 0.70; (F1-3D) avg. Precision: 0.68; AUC (F1-3D): 0.937; F1 score: 0.78 **Macular atrophy:** Mean Detection Recall: 0.91; Mean Detection precision: 0.72; F1-3D avg. Precision: 0.83; AUC: 0.970; F1 score: 0.89	NR
Wang (2022) [45]	DL	Self-attended U-Net	Attention modules	**Baseline:** Accuracy: 0.98; Sensitivity: 0.85; Specificity: 0.99; IoU: 0.77; DSC: 0.85 **Month 12:** Accuracy: 0.95; Sensitivity: 0.75; Specificity: 0.98; IoU: 0.68; DSC: 0.78	NR
Wu (2019) [46]	DL	U-Net	Probability map	AAD: 11.0; OR: 0.779; DSC: 0.87	NR
Xu (2019) [49]	DL	Stacked sparse autoencoder	NR	**DS1 with training data:** AAD (mm^2^): 0.18; AAD (%): 3.67; OR: 0.9985 **DS1 without training data:** AAD (mm^2^): 0.21; AAD (%): 4.79; OR: 0.9979 **DS2 with training data:** AAD (mm^2^): 0.48; AAD (%): 11.09; OR: 0.8455 **DS2 without training data:** AAD (mm^2^): 0.4418; AAD (%): 11.09; OR: 0.8448	NR
Zhang (2021) [56]	DL	Modified U-Net	NR	**GA approach 1:** DSC median: 0.84; DSC mean: 0.75 **GA approach 2:** DSC median: 0.83; DSC mean: 0.75	**GA approach 1:** Accuracy: 0.91; Sensitivity: 0.99; Specificity: 0.54; F1 score: 0.94; DSC median: 0.96; DSC mean: 0.91 **GA approach 2:** Accuracy: 0.94; Sensitivity: 0.98; Specificity: 0.76; F1 score: 0.96; DSC median: 0.95; DSC mean: 0.87 **iRORA/cRORA approach 1:** Accuracy: 0.89; Sensitivity: 0.90; Specificity: 0.68; F1 score: 0.94 **iRORA/cRORA approach 2:** Accuracy: 0.87; Sensitivity: 0.90; Specificity: 0.68; F1 score: 0.93

AAD (absolute area difference); AUC (area under the curve); AUPR (area under the precision-recall curve); AUROC (area under the receiver operating characteristic curve); BM (Bruch’s membrane); CAM (class activation map); CNN (convolutional neural network); cRORA (complete retinal pigment epithelium and outer retinal atrophy); DL (deep learning); DS (dataset); DSC (Dice similarity coefficient); FAF (fundus autofluorescence); FCM (fuzzy c-means clustering); FCNN (fully convolutional neural network); GA (geographic atrophy); Grad-CAM (gradient-weighted class activation map); IoU (intersection over union); iRORA (incomplete retinal pigment epithelium and outer retinal atrophy); k-NN (K-nearest neighbors); LayerCAM (layer-wise class activation map); ML (machine learning); MS-CAM (multiscale class activation map); NR (not reported); OAC (optical attenuation coefficient); OCT (optical coherence tomography); OR (overlap ratio); PPV (positive predictive value); SDC (symmetric Dice coefficient); SLO (scanning laser ophthalmoscopy); subRPE (sub-retinal pigment epithelium); TSSK-Net (teacher–student self-supervised knowledge distillation network); XAI (explainable artificial intelligence). Bold formatting is used to differentiate performance metrics across datasets, models, and approaches for clearer comparison.

## Supplementary Materials

The following supporting information can be downloaded at: https://www.mdpi.com/article/10.3390/bioengineering12050475/s1, Table S1: PRISMA 2020 checklist; Table S2: Database search terms and search strategy; Table S3: QUADAS-2* risk of bias and applicability assessment of included studies; Table S4: Assessment of AI reporting standards using CLAIM** checklist for included studies.

## Abbreviations

The following abbreviations are used in this manuscript:

AAD	Absolute Area Difference
AI	Artificial Intelligence
AIA	AI Act
AMD	Age-related Macular Degeneration
AUC	Area Under the Curve
AUPR	Area Under the Precision-Recall Curve
AUROC	Area Under the Receiver Operating Characteristic Curve
BCVA	Best-Corrected Visual Acuity
CAD	Computer-Aided Diagnosis
CAM	Class Activation Mapping
CAM*	Classification of Atrophy Meetings
CFP	Color Fundus Photography
CI	Confidence Interval
CLAIM	Checklist for Artificial Intelligence in Medical Imaging
CNN	Convolutional Neural Network
cORA	Complete Outer Retinal Atrophy
cRORA	Complete Retinal Pigment Epithelium and Outer Retinal Atrophy
cSLO	Confocal Scanning Laser Ophthalmoscopy
DL	Deep Learning
DSC	Dice Similarity Coefficient
DSS	Decision Support System
EHDS	European Health Data Space
FA	Fluorescein Angiography
FAF	Fundus Autofluorescence
GA	Geographic Atrophy
GDPR	General Data Protection Regulation
GT	Ground Truth
HIPAA	Health Insurance Portability and Accountability Act
ICTRP	International Clinical Trials Registry Platform
iORA	Incomplete Outer Retinal Atrophy
IoU	Intersection over Union
iRORA	Incomplete Retinal Pigment Epithelium and Outer Retinal Atrophy
k-NN	k-Nearest Neighbor
ML	Machine Learning
NIR	Near-Infrared Reflectance
OCT	Optical Coherence Tomography
OR	Overlap Ratio
PICOS	Population Intervention Comparator Outcome Study Design
PPV	Positive Predictive Value
PRISMA	Preferred Reporting Items for Systematic Reviews and Meta-Analyses
QUADAS-2	Quality Assessment of the Diagnostic Accuracy Studies-2
RCT	Randomized Controlled Trial
ROI	Region of Interest
RORA	Retinal Pigment Epithelium and Outer Retinal Atrophy
RPE	Retinal Pigment Epithelium
SD	Standard Deviation
SD-OCT	Spectral-Domain Optical Coherence Tomography
SS-OCT	Swept-Source Optical Coherence Tomography
WHO	World Health Organization
